# Enhanced care planning and clinical-community linkages versus usual care to address basic needs of patients with multiple chronic conditions: a clinician-level randomized controlled trial

**DOI:** 10.1186/s13063-020-04463-3

**Published:** 2020-06-11

**Authors:** Alex H. Krist, Kristen O’Loughlin, Steven H. Woolf, Roy T. Sabo, Jennifer Hinesley, Anton J. Kuzel, Bruce D. Rybarczyk, Paulette Lail Kashiri, E. Marshall Brooks, Russel E. Glasgow, Amy G. Huebschmann, Winston R. Liaw

**Affiliations:** 1 Department of Family Medicine and Population Health, Virginia Commonwealth University, One Capital Square Room 631, 830 East Main St, Richmond, VA 23219 USA; 2 Department of Psychology, Virginia Commonwealth University, Richmond, VA USA; 3 Center on Society and Health, Virginia Commonwealth University, Richmond, VA USA; 4 Department of Biostatistics, Virginia Commonwealth University, Richmond, VA USA; 5 Department of Family Medicine, University of Colorado School of Medicine, Aurora, CO USA; 6 Department of Health Systems and Population Health Sciences, University of Houston College of Medicine, Houston, TX USA

**Keywords:** Primary care, Health behaviors, Mental health, Social determinants of health, Health risk assessment, Goal setting, Health promotion

## Abstract

**Background:**

Many patients with poorly controlled multiple chronic conditions (MCC) also have unhealthy behaviors, mental health challenges, and unmet social needs. Medical management of MCC may have limited benefit if patients are struggling to address their basic life needs. Health systems and communities increasingly recognize the need to address these issues and are experimenting with and investing in new models for connecting patients with needed services. Yet primary care clinicians, whose regular contact with patients makes them more familiar with patients’ needs, are often not included in these systems.

**Methods:**

We are starting a clinician-level cluster-randomized controlled trial to evaluate how primary care clinicians can participate in these community and hospital solutions and whether doing so is effective in controlling MCC. Sixty clinicians in the Virginia Ambulatory Care Outcomes Research Network will be matched by age and sex and randomized to usual care (control condition) or enhanced care planning with clinical-community linkage support (intervention). From the electronic health record we will identify all patients with MCC, including cardiovascular disease or risks, diabetes, obesity, or depression. A baseline assessment will be mailed to up to 50 randomly selected patients for each clinician (3000 total). Ten respondents per clinician (600 patients total) with uncontrolled MCC will be randomly selected for study inclusion, with oversampling of minorities. The intervention includes two components. First, we will use an enhanced care planning tool, *My Own Health Report (MOHR)*, to screen patients for health behavior, mental health, and social needs. Patients will be supported by a patient navigator, who will help patients prioritize needs, create care plans, and write a personal narrative to guide the care team. Patients will update care plans every 1 to 2 weeks. Second, we will create *community-clinical linkage* to help address patients’ needs. The linkage will include community resource registries, personnel to span settings (patient navigators and a community health worker), and care team coordination across team members through MOHR.

**Discussion:**

This study will help inform efforts by primary care clinicians to help address unhealthy behaviors, mental health needs, and social risks as a strategy to better control MCC.

**Trial registration:**

ClinicalTrials.gov: NCT03885401. Registered on 19 September 2019.

## Contributions to the literature

The contributions to the literature are as follows:

- Enhanced care planning to address unhealthy behaviors, mental health needs, and social risks may do more to influence health than traditional medical care.
- Resources required to address health behaviors, mental health, and social risks extend beyond the healthcare setting.
- By creating a care planning tool, redefining primary care roles to function as a patient navigator, creating partnerships with community programs, and making community health workers available, primary care practices may be able to meaningfully address these complex issues.

## Background

The number of patients in the USA with multiple chronic conditions (MCC) is growing. More than one in four Americans have two or more chronic medical conditions [1]. In older populations, this increases to as many as two in three Americans. Seven of the top 10 causes of death are chronic conditions [2]. Not surprisingly, treatment of patients with MCC accounts for 75% of our national healthcare costs and 90% of Medicare spending [3]. Heart disease, stroke, diabetes, obesity, and major depressive disorder (MDD) are among the most common and manageable chronic conditions. Heart disease and stroke account for nearly one third of all deaths [4]. Diabetes is the sixth leading cause of death and the leading cause of kidney failure, amputation, and blindness [5]. More than one third of adults are obese, which shortens life expectancy [6–8] and increases the risk of other chronic diseases [9–12]. MDD is associated with higher mortality and reduced quality of life [13–15], is the leading cause of disability in adults in high-income countries [16, 17], and impairs individuals’ ability to manage their health.

### Role of health behaviors, mental health, and social needs in MCC

Physical inactivity, poor nutrition, tobacco use, and overconsumption of alcohol cause much of the illness, suffering, and early death related to MCC [18]. Unhealthy behaviors are ubiquitous in the USA—only 21% of adults exercise adequately, 40% eat insufficient vegetables and fruit, 15% smoke, and 20% binge drink an average of four times per month [19–22]. Unhealthy behaviors increase cardiovascular risks, cause elevated blood glucose, exacerbate diabetes-related complications, cause weight gain, and impair mood, ultimately leading to uncontrolled chronic conditions. Given the harms of unhealthy behaviors and the benefits of behavioral counseling, the US Preventive Services Task Force (USPSTF) recommends screening and counseling for healthful diet, physical activity, obesity, smoking, and alcohol misuse [23–27]. Mental illnesses and psychosocial distresses are both chronic conditions and—as in the case of stress and anxiety—are contributors to poor MCC control. Distressed patients are less likely to seek medical care, adhere to care plans, or maintain healthy behaviors. Likewise, patients with MCC have lower physical and social functional status, less favorable mental well-being and health perceptions, and greater pain than do patients without chronic conditions [28]. Mental illness, psychosocial distress, and uncontrolled MCC reinforce and exacerbate each other [29]. Social needs—defined here as housing, food, transportation, finances, employment, education, and safety—also influence health outcomes [30–36]. They contribute to health inequities, higher costs, and overutilization of health services. Clear evidence shows that social determinants of health have a greater impact on morbidity, mortality, and quality of life than medical care itself [30, 34–42]. The National Academy of Medicine (NAM), the Centers for Medicare & Medicaid Services (CMS), and the World Health Organization have called for primary care and public health to address social needs [35, 43–45].

### Addressing health behaviors, mental health, and social needs

**Fig. 1:**
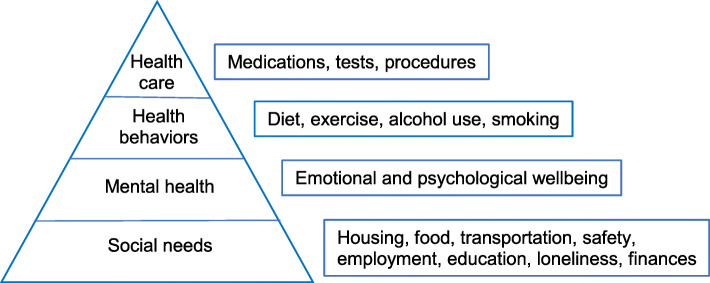
Hierarchy of social, mental health, health behavior, and healthcare needs. Patients struggling to address more basic needs like social risks, mental health needs, or unhealthy behaviors will have more difficulties engaging in their health care

The chronic care model informs how communities and healthcare systems can better address MCC [46–49]. It describes how self-management support, delivery system design, decision support, and clinical information systems can lead to a prepared, proactive practice team and an informed and activated patient (the latter specified as a required element to achieving improved health outcomes). While educational programs and efforts by clinicians to engage patients can help produce an “informed and activated patient,” patients cannot participate fully in their health care if they are struggling to meet basic needs. Patients facing these challenges are likely less capable of participating in a higher-level functional activity, such as health care (Fig. 1).

Effectively addressing health behaviors, mental health, and social needs can be more challenging than traditional medical services. Health behavior counseling requires dozens of hours of face-to-face contact by a multidisciplinary team over many months [50, 51]. The mental healthcare delivery system is not well integrated with medical care [52]. The healthcare system is only beginning to consider its role in addressing the complex problems patients face in getting stable housing, food security, employment, loneliness, and education [53]. Social needs cover a wide range of problems, each involving different sectors, agencies, and networks of community organizations. Community programs, social service agencies, public health departments, policymakers, and other stakeholders have been working arduously to address these needs. For example, many community recreation centers offer diet and exercise programs, nutritional counseling, group classes, and chronic disease programs. Policymakers are considering “health in all” programs, redesigning public housing, enacting laws to curb smoking and unhealthy diet, and supporting social services and mental health. Health departments, community service boards, housing authorities, food shelters, and other entities are addressing poverty, job training, unemployment assistance, transportation services, violence prevention, and advocacy. These activities are generally outside the purview of traditional primary care training and activities. An unmet need is for primary care clinicians to find their place among those efforts—a pathway for clinicians to contribute.

Primary care clinicians have need for systems to connect patients with these resources because of the frequency with which they encounter behavioral, mental health, and social needs in routine practice. Virginia Commonwealth University (VCU) led a national study to field *My Own Health Report (MOHR)*, a health risk assessment and care planning tool administered in nine diverse settings to screen for 13 health behavior and mental health needs. Patients reported an average of 5.8 needs [54–56]. The majority had six or more needs, and less than 1% had no needs. In an ongoing study asking patients about social needs in 12 practices that serve an affluent Northern Virginia population, high rates of basic social needs were reported—71% had at least one [57, 58]. Although addressing every need is not feasible, 3–20% of patients wanted clinician help with a limited set of prioritized needs. A patient-centered approached guided by patient interest in addressing needs is more manageable and likely to help patients.

### Scientific premise

Health systems are increasingly held accountable for health outcomes and reducing costs. Recognizing the importance of social determinants of health, health systems are developing models for connecting patients with programs addressing behavioral health and social needs. Hospitals, emergency departments, and other providers are partnering with social services, public health, and community-based organizations. Models are proliferating across the country, including Hennepin Health, the Chicago-based Community Rx, the Michigan Public Health Institute Pathways to Better Health Community Hub, the American Hospital Association, Practical Playbook, and the EveryONE Project [45, 59–61]. These projects show the importance of cross-sector partnerships, data systems to bridge clinical and community care, and the need for community health workers and navigators [45]. In 2017, the CMS catalyzed the movement by funding 31 communities to develop and test new payment and service models, termed Accountable Health Communities (AHCs), which build clinical-community partnerships [62]. Private payers, employers, and the public health sector are further supporting these efforts, resulting in growing national momentum for change.

While these changes are exciting, they have two major limitations. First, few are being evaluated in randomized controlled trials (RCTs). The CMS AHC program utilizes a dissemination design, focused on maximizing the number of participants and discouraging comparison groups. This limits their ability to demonstrate efficacy. Second, few have a defined role for primary care; most are designed for use by health systems in centralized care settings, such as emergency departments or hospitals. This hospital-centric focus is reflected in the CMS-mandated outcome of AHCs: reduction in hospital and emergency department admissions.

Primary care practices, in contrast, are distributed across communities and need innovative models for tapping into such systems. Few primary care practices can build their own infrastructure. An innovative solution is to establish practice systems that take advantage of the hospital- and health system-based programs that are proliferating across the country. To do this, primary care practices need (1) a system to help patients relay their needs and collaboratively create care plans that incorporate their values, preferences, and personal, social, and clinical context, and (2) a system to connect patients with community resources to help patients achieve their care plan goals.

## Methods

**Fig. 2:**
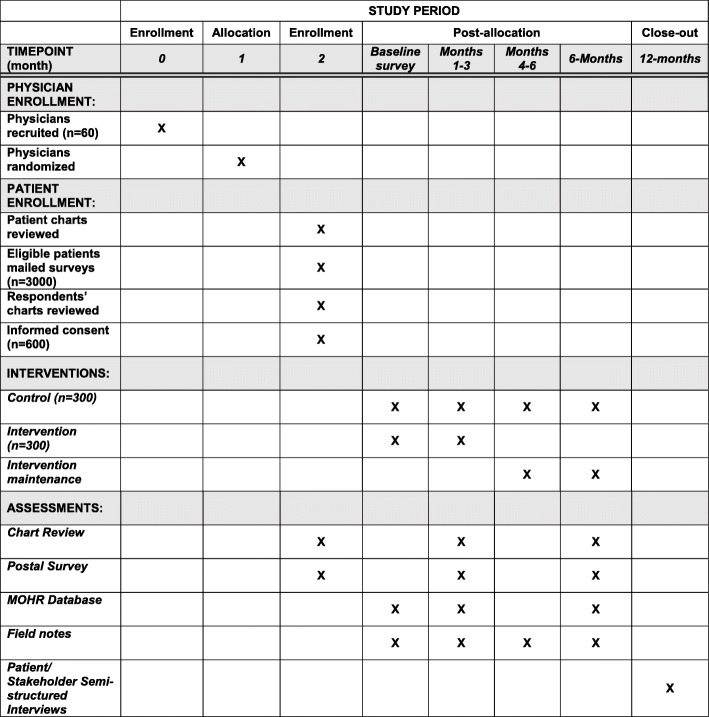
Standard Protocol Items: Recommendations for Interventional Trials (SPIRIT) schedule of assessments and interventions. Sixty clinicians will be randomized to the intervention or control condition in a 1:1 ratio. Fifty patients with chronic conditions will be randomly selected to receive the baseline screening survey. Among respondents, 300 patients (10 per clinician) will be randomly selected for study participation. The intervention will include a 3-month intensive care planning and support period and a 3-month maintenance support period. Control patients will continue with usual medical care. Clinicians and patients will be recruited quarterly between December 2019 and December 2021, allowing for approximately 40 patients each quarter to receive intensive care planning support

This is a clinician-level cluster RCT to study the effectiveness of enhanced care planning to manage MCC. We will use a hybrid implementation-effectiveness design to understand how the intervention is implemented and why it is successful. Patients with two or more uncontrolled MCC will receive the intervention or continue usual care, as depicted in Fig. 2. The intervention consists of two components: (1) assessment of unhealthy behaviors, mental health needs, and social needs and the creation of a patient-centered enhanced care plan, and (2) clinical-community linkages to create care teams to assist patients with their care plan. Outcomes will include intervention implementation and effectiveness compared to usual care in controlling MCC and patient-reported physical, mental, and social well-being. All analyses will be patient-level and intention to treat. A mixed methods contextual assessment will assess the person, family, community, and healthcare system factors that influence the intervention’s effectiveness [63]. This study has been approved by the VCU Internal Review Board (IRB HM20011992) and contains no more than minimal risk to participants. The risks are limited to breaches of privacy and confidentiality.

### Specific aims

#### Specific aim 1 (implementation)

Aim 1 is to evaluate the implementation and implementation outcomes of enhanced care planning and clinical-community linkages for care teams to better address MCC in primary care. From MOHR data, chart review, patient navigator and community health worker (CHW) field notes, and patient postal survey data, we will measure four implementation metrics:

1. *Sub-aim 1a.* The percentage of identified patients with MCC who complete an enhanced care plan
2. *Sub-aim 1b.* The prevalence of identified patient health behavior, mental health, and social needs
3. *Sub-aim 1c.* The goals that patients prioritize and how they want to address them
4. *Sub-aim 1d.* The type, intensity, and follow-up of care team support provided to patients to address identified needs and concordance of support with patient-identified goals.

#### Specific aim 2 (effectiveness)

Aim 2 is to compare in a pragmatic clinician-level RCT with 60 clinicians and 600 patients the effectiveness of enhanced care planning and clinical-community linkages for improving control of MCC and patient-reported physical, mental, and social well-being relative to usual care. From electronic health record (EHR) data and patient surveys, we will determine whether patients randomized to the intervention have better control of their chronic conditions and score better on the physical, mental, and social domains of the EuroQol five-dimension instrument (EQ-5D) than do control patients who receive usual care.

- *Hypothesis 1.* Compared to control patients, intervention patients will have at least a 10% overall reduction in the number of uncontrolled chronic conditions (e.g., hemoglobin A1c above 8.0, blood pressure above 150/90 or 140/90 mmHg per guidelines, not taking a statin or aspirin if 10-year cardiovascular risk is above 10% and is otherwise indicated, eating fewer than five servings of vegetables and fruits per day, exercising less than 150 min per week, moderate or severe depression) when measured 6 months and 2 years after enrollment.
- *Hypothesis 2.* Compared to control patients, intervention patients will report better physical, mental, and social health as measured by the EQ-5D profile 6 months and 2 years after enrollment.

#### Specific aim 3 (context)

Aim 3 is to understand the person, family, community, and care system contextual factors that influence the implementation and effectiveness of the intervention in addressing patient needs and controlling MCC. From EHR data, patient surveys, field notes, and a series of interviews (*n* = 64) with selected patients, clinicians, community care providers, and program leaders, we will assess the types of support patients receive, whether the support was adequate to meet needs, additional support needed for success, factors facilitating or hindering success, and necessary elements for sustainability.

### Study sample

Beginning December 2019, we will recruit clinicians from Virginia Ambulatory Care Outcomes Research Network (ACORN) primary care practices located in the Greater Richmond Region. ACORN has academic affiliations with nearly 500 primary care practices, 53 of which are located in the Greater Richmond Region. Practices range in size from 2 to 18 providers and operate under diverse ownership and insurance models.

Sixty clinicians will be randomly and equally (1:1) allocated between the intervention and control conditions using stratified randomization with matching achieved by randomly allocating clinicians within each of the four cross-classifications of age (less than 50, 50 or older) and sex (male or female). Allocation will be conducted by the study biostatistician, who will use the R statistical software to generate random numbers (between 0 and 1) for each clinician, allocating to the intervention for numbers greater than or equal to 0.5, and otherwise allocating to the control. Patients and chart auditors will be blinded to allocation, though clinicians will not be blinded due to the nature of the intervention. From the EHR, we will obtain a list of all patients seen the prior year including their demographic information (age, sex, race, ethnicity, insurance type), diagnoses, and vital signs (body mass index [BMI] or height and weight). After identifying all eligible patients, we will use stratified sampling to randomly sample approximately 50% white and at least 30% black, 10% Asian, 10% multiracial, and 10% Hispanic patients (of any race); we will purposefully oversample Asian and Hispanic patients to ensure representative samples. Eligibility criteria are age 18 and older and the presence of two or more chronic conditions (cardiovascular disease, hypertension, hyperlipidemia, diabetes, obesity, MDD).

In March 2020, we will randomly select 50 patients from each intervention and control clinician’s panel and mail a baseline survey. We will review the charts of the survey respondents and assess their survey responses to identify if their MCC are uncontrolled. From this sample, we will randomly select 10 patients with at least one poorly controlled MCC for study inclusion. Study research coordinators will contact patients to arrange an in-person or virtual meeting to consent the patients, enroll them in the study, and create the patient’s initial MOHR account. On the consent form, participants will be asked if they agree for their data to remain part of the study (unless requested otherwise) should they choose to withdraw from the trial. Participants will also be asked for permission for the research team to share relevant data with the practices taking part in the research or from regulatory authorities, when relevant. The consent form states that information collected as part of the study will not be used or distributed for future research studies. This trial does not involve collecting biological specimens for storage.

### Intervention and control conditions

#### Intervention condition

**Fig. 3:**
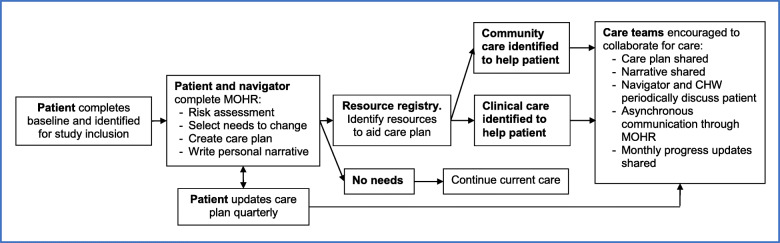
Overview of enhanced care planning intervention. The intervention includes creation of an enhanced care plan (MOHR), redefined (patient navigator) and new (community health worker) care roles, a resource registry, and linkage support to community programs (e.g., sharing care plans, communication tools)

Our intervention consists of two components: enhanced care planning and clinical-community linkages (Fig. 3). Intervention clinicians will identify a patient navigator (e.g., a nurse, medical assistant, social worker, or behaviorist). The clinicians and navigator should have daily contact. Additionally, we will fund one CHW to help coordinate care across clinical and community settings, and the patient navigators and CHW will work closely together. We will also identify a central contact person for the key community programs. While intervention patients will continue to receive usual care in addition to the intervention, it is possible for the intervention to alter care, including medications, testing, or other interventions for the treatment of MCC.

#### Enhanced care planning

Intervention clinicians will be given a list of 10 patients with MCC who meet the study inclusion criteria. Patients will be asked to complete an enhanced care plan using MOHR with the patient. A private version of MOHR requiring sign-in with expanded functionality will be used for this study. Additions include multiple user types (patient, clinician, navigator, CHW, community program), dynamic repeated use, and communication tools. MOHR begins with a health behavior, mental health, and social needs assessment for 18 domains (Table 1) [55]. Through programmed logic based on national guidelines, the system identifies patient needs and asks which ones he/she would like to change. Patients are directed through the creation of SMART (Specific, Measurable, Actionable, Realistic, and Time-based) goals [64, 65]. With the assistance of the clinician, patient navigator, and CHW, these SMART goals are adapted to become a care plan including specific steps and clinical and community programs to assist patients in achieving their goals. Next, patients are encouraged to complete a patient narrative describing important characteristics about who they are as a person. This information will help the care team to better guide care for them. Patients will be asked to update their care plans regularly and provide feedback on progress. For those who do not update progress, or for whom issues are raised, the patient navigator will call the patient and verbally update progress or address issues.

**Table 1:** MOHR health behavior, mental health, and social needs content

**Health behaviors**	
- Daily fruit and vegetable intake	
- Weekly fast food intake	
- Daily soda intake	
- Weekly exercise	
- Smoking habits	
- Alcohol use	
- Illegal drug use	
**Mental health**	
- Depression	
- Anxiety	
- Stress	
**Social needs**	
- Financial status	
- Employment	
- Food security	
- Transportation	
- Housing	
- Dental care	
- Safety	
- Loneliness	

#### Clinical-community linkage

Building on our framework for successful community-clinical linkages, the intervention will include five main components. First, we will provide all clinicians and patient navigators a resource registry. As part of preparatory work for the study, we will identify all the local resources to address the potential health behavior, mental health, and social domains. This will be updated regularly by the patient navigators and CHW. Second, clinicians will identify a patient navigator, and we will employ one CHW. The navigator will be the primary clinical contact, helping with creating care plans, checking on progress, and thinking about programs to help patients. Patient navigators can refer patients to the CHW, who will serve as a primary contact for patient referral to and participation in community programs. The CHW can ensure patients follow up with community programs, get the assistance they need, and communicate progress with the clinical care team. Third, we will configure MOHR to share information (care plans, patient narrative, and patient progress) across the clinical and community care team members. All care team members will have MOHR accounts to view their patient panel and each individual patient’s progress. Fourth, we will configure MOHR to allow for asynchronous communication (i.e., messaging). Patients will be able to initiate a message to any care team member or vice versa. Finally, as patients provide updates to their care plan through MOHR, all care teams will be alerted on patient progress.

#### Control condition

Clinicians randomized to the control condition will continue to provide “usual care.” This includes current non-systematic assessment of health behaviors, mental health needs, and social needs. Neither clinicians nor patients will be eligible to receive CHW support or have access to the resource registry. Control clinicians will be blinded as to which patients are included in the study. At the end of the study, we will share with control clinicians our lessons learned, access to MOHR, and lists of useful community resources. As a result, we do not expect the usual care group to have altered treatment as a result of study participation.

### Criteria for discontinuing or modifying allocated interventions

Clinicians and patients can withdraw from the study at any time. Leaving the study will not affect ongoing medical care. There will be no specific clinical or social criteria for discontinuing or modifying allocated interventions.

### Data collection

To address the questions in our three specific aims, we will use five data sources: MOHR, chart review, patient navigator and CHW field notes, patient postal survey, and patient semi-structured interviews.

#### MOHR database

The MOHR database will be the central data source to assess aim 1. The MOHR database manager will transfer data to the VCU database manager quarterly to allow the research team to provide participating clinicians feedback about progress with creating care plans. Data elements transferred will include the patient study ID; specific health behavior, mental health, and social needs; the specific needs patients want to address; patient- and clinician-generated care plans; patient narratives; and whether and how care plans are modified over time. Additionally, patients will be asked to complete the MOHR health behavior, mental health, and social risks questions once enrolled, at 6 months, and at 12 months. Responses will be used to analyze improvements in aim 2. All data will be stored in MOHR and transferred as structured categorical variables, aside from care plans and patient narratives, which will be free text in the patient’s words.

#### Patient navigator and CHW field notes

We will ask the patient navigators and CHW to record data about patient interactions, needs addressed, how needs are addressed, whether and how patients accessed services, and problems accessing services. We will create a structured online intake within MOHR that navigators and CHW can use during patient interactions, which will longitudinally show past entries to further facilitate care.

#### Chart review

In year 1, we will use the EHR database to identify patient participants. To assess outcomes for aims 1 through 3, we will conduct a manual chart review after intervention completion. Using a structured Excel template, chart abstracters will record for each study patient the patient study ID; age; sex; race-ethnicity; insurance type; active diagnoses; all dates and values for A1c, blood pressure, height, weight, BMI, and Patient Health Questionnaire (PHQ)-2/9 score; cholesterol and blood pressure medication use (and date started if started during intervention period); and 10-year pooled cohort equation cardiac risk. Additionally, abstracters will record any documented referrals to address patient needs, any text documentation about clinician-delivered counseling, and documented successes or challenges with addressing patient’s needs.

#### Patient postal survey

To assess outcomes that can only be determined by patient report, we will mail a survey to all intervention and control patients (*n* = 600) 6 months and 2 years after identification and randomization. Additionally, to select the patient study sample, we will mail a baseline survey to 50 patients with MCC for each clinician (*n* = 3000 total). For each survey, we will optimize the response rate by using a modified Dillman method [66, 67], mailing surveys on practice stationery and in practice envelopes, including a personal note from the patient’s clinician, and providing a $2 incentive [68]. The baseline survey will include the National Cancer Institute (NCI) fruit and vegetable screener to determine the average servings per day [69], a two-question “exercise vital sign” check to calculate the minutes per week of exercise [69, 70], and the EQ-5D [71, 72]. The 6-month and 2-year surveys will include similar content plus questions about what support patients received, challenges addressing needs, and whether they still have the need. Survey responses will be linked to MOHR responses to measure improvements in aim 2.

#### Patient and stakeholder semi-structured interviews

We will identify and recruit 48 patients for semi-structured interviews—16 with a health behavior need, 16 with a mental health need, and 16 with a social need—within each group, with half who reported their need persists and half who reported it resolved. We will develop an interview guide that queries patients about the following: (1) specific needs, (2) care plans they developed with their care team, (3) types of support they wanted to receive and did receive, and (4) the person, family, community, healthcare system, community program, and policy factors that aided or hindered their success. We will also interview stakeholders from community programs, eight who provided support to more intervention patients and eight who provided support to few, as well as eight clinicians and patient navigators whose patients were in the highest quartile for improvement and eight whose patients were in the lowest quartile (according to aim 2). These interviews will review the patient cases in which they participated and solicit their perspectives on factors aiding and hindering success. Interviews will occur 1–3 months following intervention completion. Interviews will be conducted in person, recorded, and transcribed.

### Analytic plan

An overview of our data collection methods is presented in Table 2. Outcomes span three domains: implementation, effectiveness, and context. These outcomes will provide an understanding of the intervention, capacity of primary care and communities to do care planning and participate in the intervention, contextual factors that influence outcomes, and potential for dissemination.

**Table 2:** Overview of data collection methods and analysis

Aim	Data sources	Analysis
**Aim 1, Implementation:** To evaluate how the intervention is implemented	• *MOHR database* to identify which patients complete care plans, patient needs and wants • *Field notes*, *patient survey* to identify the types of support provided to patients to address identified needs	• Percentage of intervention patients who complete a care plan (generalized linear mixed models) • Frequency of patient needs and needs patients want to address (generalized linear mixed models) • Support provided patients and consistency with patient wants (generalized linear mixed models)
**Aim 2, Effectiveness:** To compare the effectiveness of intervention versus usual care for control of MCC and patient-reported physical, mental, and social health	• *Chart review* to measure change in A1c, blood pressure, and statin and aspirin use • *Patient survey* to measure diet, exercise, depression, and EQ-5D domains at baseline, 6 months, and 2 years	• Percentage of patients with controlled MCC for intervention patients versus usual care (generalized linear mixed models) • Pre-post change in EQ-5D domains for intervention patients versus usual care (linear mixed-effects models)
**Aim 3, Context:** To understand the contextual factors that influence intervention implementation and effectiveness	• *Patient survey* to identify success in addressing needs • *Semi-structured interviews* with patients, patient navigators, CHW, community providers, and program leaders	• Identification of common themes influencing success in addressing needs and controlling MCC (immersion/crystallization analysis)

#### Implementation outcomes

Aim 1 addresses implementation outcomes. Sub-aim 1a, the percentage of identified patients with MCC who complete an enhanced care plan, will be defined as all patients assigned to the intervention condition to complete the MOHR assessment and develop a care plan. For sub-aim 1b, the frequency of identified patient’s needs, the MOHR health risk assessment has built-in logic to define unhealthy behaviors, psychosocial distress, and social needs. The logic is based on national standards defined by the Dietary Guidelines for Americans [73], thePHQ-9 [74, 75], the Generalized Anxiety Disorder (GAD) questionnaire [76, 77], the Alcohol Use Disorders Identification Test (AUDIT-C) [78, 79], the Drug Abuse Screening Test (DAST-10) [80], and 10 domains of social needs identified by the National Academy of Medicine (NAM) [81, 82]. For sub-aim 1c, the needs patients want to address are identified in MOHR. How they want to address the needs will be derived from the care plans patients create in MOHR. As part of the qualitative review process, we will group how patients want to address needs into categories. Both patients’ needs and how they want to address them can change over time. For sub-aim 1d, we will derive the type and intensity of support provided to address patients’ needs from field notes, chart abstraction, and patient postal survey. This will also be categorized during the qualitative review process. Concordance with what patients want and what is done will be assessed by both the research team (concordance with assigned categories of support) and the patient (asked on patient survey if the support received was what they wanted).

To evaluate how the intervention is implemented, we will calculate the frequencies and percentages for the implementation measures overall and adjusted by specific patient characteristics (e.g., patient sex, age, race and ethnicity, insurance type), clinician, and practice. Adjusted percentages of these measures will be estimated using the generalized linear mixed model (GLMM) framework, with fixed effects for patient characteristics, and where the outcomes are a binary or multinomial indicator for the relevant implementation measure (i.e., whether a patient with MCC completed a care plan, identified a need, and type and intensity of care team support provided). In these models if a patient characteristic is significant at the 5% level, multiple comparisons will be conducted to examine differences in the implementation percentage based on various levels of measurement.

#### Effectiveness outcomes

Aim 2 addresses effectiveness outcomes—constituting the study’s primary outcome. It includes seven intermediate outcomes that reflect better control of MCC: controlled A1c and blood pressure, appropriately taking aspirin or a statin, healthy diet and exercise, and depression severity of mild to none. We selected these intermediate outcomes based on current guidelines (Table 3). This aim is our primary outcome and the basis for our power calculation. The A1c, blood pressure, and aspirin or statin use will be measured by EHR chart abstraction; other outcomes will be measured at 6 months and 2 years from the patient survey. For each, we will measure the change from baseline to 6 months and 2 years. For EHR measures, we define baseline as the most recent value prior to starting the study and the 6-month and 2-year values as the most immediate value after the measure date. For each patient eligible for the MCC control outcomes, we will define the patient categorically as controlled or uncontrolled. We will also calculate change from baseline to 6 months and baseline to 2 years for each of the five question topics included on the EQ-5D: mobility, self-care, usual activities, pain/discomfort, and anxiety/depression. Each domain is scored per defined scoring procedures.

**Table 3:** Defining controlled multiple chronic condition outcomes

MCC	Eligible population	Controlled MCC
Diabetes	Diabetic patients	A1c < 8
Blood pressure [83]	18–59 years or any age, diabetes/kidney disease	Blood pressure ≤ 140/90
	60 years and older, no diabetes/kidney disease	Blood pressure ≤ 150/90
Cardiovascular prevention [84, 85]	40–75 years and ≧ 10% cardiovascular risk	Taking a statin
	50–69 years and ≧ 10% cardiovascular risk	Taking an aspirin
Healthy behaviors [73]	All patients	≥ 5 servings vegetables and fruit per day
	All patients	≥ 150 min exercise per week
Depression [86]	Patients with MDD	None to mild depression on PHQ-2

To compare the effectiveness of intervention versus usual care, we will use hierarchical GLMMs to compare over time and between groups the unadjusted change in mean number of uncontrolled MCC and patient-reported measures (EQ-5D). These models will include the number of uncontrolled MCC (modeled as a negative binomial count to account for potential overdispersion) and the continuous score from the EQ-5D profile (modeled as a continuous score), a two-level fixed-group effect (intervention or control), a three-level fixed-time effect (baseline, 6 months, and 2 years), a group-time interaction fixed effect, a patient-level random effect to account for repeated measurements over time, and both clinician-level and practice-level random effects to account for clustering of patients within both clinicians and practices. We use a difference-in-differences approach to measure effectiveness by comparing change from baseline to 6 months and baseline to 2 years between the two groups. This would account for any baseline differences between intervention and control groups. We will examine the effect of patient, clinician, and practice characteristics on effectiveness by (1) estimating adjusted comparisons and (2) searching for possible effect moderation. Adjusted comparisons will be conducted by simultaneously adding all patient (sex, age, race and ethnicity, insurance type), clinician, and practice characteristics into the mixed-effect models as fixed effects with no interactions between them and either of the group, time, and group-time interaction terms. Moderation of the treatment effect will be examined by including each patient characteristic into the model separately, where the measure is included as a fixed effect and is also interacted with the group, time, and group-time interaction effects [87].

#### Contextual outcomes

Aim 3 addresses contextual outcomes. The contextual assessment is framed around multiple levels of key contextual factors and subcategories that the Agency for Healthcare Research and Quality (AHRQ) Multiple Chronic Conditions in Context initiative identified as influential on MCC [63]. Our goal is to understand person, family, community, and healthcare system factors that influence outcomes in addressing needs and/or improving MCC. Outcomes will be derived from the qualitative evaluation of interviews and supplemented with multivariate analyses of structured data from MOHR, patient surveys, and chart reviews.

To understand contextual factors that influence intervention implementation and effectiveness, we will use the following strategies. For each patient, we will aggregate transcripts from semi-structured patient and clinician interviews, text abstracted from charts, survey responses, action plans, personal narratives, provided support, and outcomes. This will create a series of 48 patient and 16 “case studies” to analyze. We will use a grounded theory and an immersion/crystallization process to identify key themes across the series of case studies [88–94]. A codebook will be created, combining emergent and a priori themes derived from the interview guide, chart abstraction template, and surveys. Two investigators will independently code each case study. Case studies will be analyzed separately and in aggregate. When new themes emerge during the coding process, they will be discussed by the team and added to the codebook. Investigators will regularly meet throughout the coding process, and any disagreement will be resolved by consensus including a third investigator. The team will code transcripts and organize data using Atlas.ti 7.5.

### Sample size

The following power calculations are based on Murray (1998) and Donner and Klar (2000) [95, 96] to account for varying treatment effectiveness between clinicians due to (1) clinician-based randomization and (2) nesting of patients within clinicians. To account for not being able to measure 6-month and 2-year outcomes in some patients (e.g., because they move, change practices, etc.), we aim to enroll 10 patients from each of 2 clinicians from 30 practices, and conclude with data on 8 patients per clinician, for a total of 480 patients. Assuming an intra-class correlation (ICC) of 0.05, a 5% type I error rate, and a between-patient standard deviation in the number of uncontrolled MCCs of 1.0 (aim 2), we will have sufficient power. This provides 80% power to declare a 0.26 decrease in the mean number of uncontrolled MCC in the intervention group significantly different from a 0-unit change in the control group. The 0.26 decrease in mean number of uncontrolled MCC correlates with the hypothesized 10% improvement in aim 2. This goal was selected as a clinically meaningful improvement for patients.

### Trial status and monitoring

**Fig. 4:**
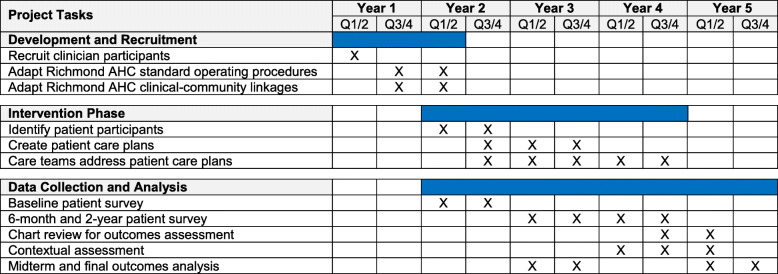
Proposed project timeline. The project began March 2019 and will continue through February 2024

The overall study timeline is shown in Fig. 4. Funded in March 2019 by the AHRQ (1R01HS026223-01), preparation for recruitment has begun. The study is expected to begin recruitment of clinicians in August 2020 and patients in October 2020. Recruitment will continue over a 2-year period and be completed in August 2022. A data safety monitoring board (DSMB) will be assembled at the start of clinician recruitment and will meet annually to perform an annual audit that will include monitoring study progress, participant safety, and interim results. If the DSMB has any study concerns, they will be able to terminate the study early. The DSMB will include a biostatistician, a primary care clinician researcher, and a social worker. The DSMB will be an independent body free from competing interests. Patients, clinicians, patient navigators, and the CHW will be able to report adverse events to the investigators or the VCU IRB at any point. All reported adverse events will be shared with the VCU IRB and the DSMB. Any protocol modifications will be updated on ClinicalTrials.gov and submitted to the VCU IRB.

Findings from this study will be published in medical journals and presented at medical conferences. Participating clinicians and patients will be given summaries of results prior to publication, and feedback will be incorporated in publications. The investigators will have no restrictions in what they can publish or present. This article presents protocol version 1.2 of the study, which was finalized September 15, 2019.

## Discussion

Patients with MCC have a range of needs that extend beyond traditional medical care, including behavioral, mental health, and social needs. While primary care does its best to address these needs, few practices can undertake a systematic approach without broader health system and coordinated community support. Fortunately, communities and health systems are investing in new models of care to address these needs.

This study will test an innovative model for how primary care clinicians can participate in these systems and measure whether patients with uncontrolled chronic conditions benefit from improved access to these resources. Specifically, it will delineate how both an enhanced care planning approach and community-clinical linkages support are implemented, the effectiveness relative to usual care, and contextual factors that influence outcomes. Findings will help inform efforts by primary care clinicians to participate in the growing number of AHC-like systems as a strategy to better control MCC. If effective, clinicians participating in this study would ideally continue to provide the supports we are studying for their participating patients and even extend the approach to others with MCC.

This study has several limitations. First, by including only patients who return the baseline survey, our design introduces a respondent bias. Respondents may have different needs, but we need baseline responses to assess uncontrolled MCC for study inclusion. Second, doing a clinician-level rather than practice-level randomization runs a risk for contamination between intervention and control groups. However, given the number of practices available, a practice-level randomization is not feasible. Most importantly, the intervention is designed to limit contamination. Use of MOHR will require user accounts, which will only be given to intervention clinicians and clinical navigators, and CHWs will agree to only provide services for intervention clinicians. While control clinicians may learn about existing community resources, they will not have support to coordinate care, which mimics current usual care. The harms to potential study participants are minimal. The main risks are breaches to confidentiality and privacy and potential stress and anxiety as intervention patients address their health behavior, mental health, and social needs. We will use proven protocols to protect privacy and confidentiality.

Our study includes critically needed innovations, including care planning for MCC, addressing domains of health behaviors, mental health, and social needs; letting patients choose the needs they wish to address; evaluating outcomes in an RCT to assess causality; and conducting a contextual assessment that can inform similar interventions in other communities. Most importantly, the key innovation is to test a generalizable model for primary care to become involved in the growing number of national initiatives designed to address the root causes of uncontrolled disease. Primary care is in a unique position to meaningfully contribute by identifying individuals who are most in need and most likely to benefit, facilitating coordination across a range of settings, tailoring care to the individual, providing ongoing motivation and encouragement, and acting as an advocate for the patient. The strengths of primary care—risk assessment, patient-centered care, coordinating care, transitions of care—have not been fully realized in addressing health behaviors, mental health, and social needs. Finding a solution would be a “win-win-win” for primary care, social service programs, and patients: it helps primary care address challenging issues that have historically been resistant to change, helps service providers to better deliver programs, and addresses patient needs.

Primary care provides great opportunity for addressing the needs of patients with MCC. Unhealthy behaviors, mental health challenges, and social needs contribute greatly to uncontrolled MCC, and high rates of needs are consistently encountered in these settings. This study will break new ground by testing an innovative model to address MCC, with primary care clinicians at the forefront. The methods being tested build off community care initiatives and leverage existing resources. As such efforts proliferate in communities across the country, this approach—if effective—could have widespread applicability and impact.

## Abbreviations

ACORN: Virginia Ambulatory Care Outcomes Research Network
AHC: Accountable Health Community
AHRQ: Agency for Healthcare Research and Quality
BMI: Body mass index
CHW: Community health worker
CMS: Centers for Medicare & Medicaid Services
EHR: Electronic health record
GLMM: Generalized linear mixed model
ICC: Intra-class correlation
MCC: Multiple chronic conditions
MDD: Major depressive disorder
MOHR: My Own Health Report
NAM: National Academy of Medicine
NWD: Virginia No Wrong Door
PBRN: Practice-based research network
RCT: Randomized controlled trial
SMART: Specific, Measurable, Actionable, Realistic, and Time-based (goals)
USPSTF: US Preventive Services Task Force
